# Aspirin sensitivity of *PIK3CA*-mutated Colorectal Cancer: potential mechanisms revisited

**DOI:** 10.1007/s00018-022-04430-y

**Published:** 2022-07-02

**Authors:** Daniella C. N. Hall, Ralf A. Benndorf

**Affiliations:** Department of Clinical Pharmacy and Pharmacotherapy, Institute of Pharmacy, Martin-Luther-University Halle-Wittenberg, Kurt-Mothes-Str. 3, 06120 Halle (Saale), Germany

**Keywords:** Acetylsalicylic acid, PI3 kinases, AKT, PKB, mTOR, Cyclooxygenases, COX, Wnt, *β*-catenin, Tumor Biology, Prostaglandin, NSAID

## Abstract

*PIK3CA* mutations are amongst the most prevalent somatic mutations in cancer and are associated with resistance to first-line treatment along with low survival rates in a variety of malignancies. There is evidence that patients carrying *PIK3CA* mutations may benefit from treatment with acetylsalicylic acid, commonly known as aspirin, particularly in the setting of colorectal cancer. In this regard, it has been clarified that Class IA Phosphatidylinositol 3-kinases (PI3K), whose catalytic subunit p110α is encoded by the *PIK3CA* gene, are involved in signal transduction that regulates cell cycle, cell growth, and metabolism and, if disturbed, induces carcinogenic effects. Although PI3K is associated with pro-inflammatory cyclooxygenase-2 (COX-2) expression and signaling, and COX-2 is among the best-studied targets of aspirin, the mechanisms behind this clinically relevant phenomenon are still unclear. Indeed, there is further evidence that the protective, anti-carcinogenic effect of aspirin in this setting may be mediated in a COX-independent manner. However, until now the understanding of aspirin’s prostaglandin-independent mode of action is poor. This review will provide an overview of the current literature on this topic and aims to analyze possible mechanisms and targets behind the aspirin sensitivity of *PIK3CA*-mutated cancers.

## Introduction

According to the GLOBOCAN 2020 study, cancer is a leading cause of death in industrialized countries and in most developing countries, accounting for nearly 10 million deaths worldwide [1]. For instance, according to recent estimates, every third death in Germany among people over the age of 45 is cancer-related. In addition, 1.52 million people suffering from cancer had to be treated in hospital in 2018 [2]. Colorectal cancer (CRC) is the third most common and the second most deadly cancer being responsible for approximately 10% of all cancer-related deaths worldwide [1, 3]. Known risk factors for the development of colorectal carcinoma include familial and genetic predisposition, chronic inflammatory bowel diseases as well as a diet low in fiber and vegetables, obesity, and lack of exercise [4].

For the pathogenesis of colorectal carcinoma, the so-called adenoma carcinoma-sequence is assumed. It is supposed that the majority of colorectal carcinomas develop in a multi-stage process (sequence) of genetic changes from initially benign tumors (villous and tubular adenomas) of the colon mucosa. The malignant transformation of the cells occurs gradually via mutations of various tumor suppressor and proto-oncogenes, such as *APC (adenomatous polyposis coli), KRAS (kirsten rat sarcoma virus),* and *p53 (also known as TP53, tumor protein 53)*, and subsequently leads to uncontrolled proliferation of the enterocytes and promotes invasive growth and metastasis of the degenerated cells to regional and distant lymph nodes and organs, such as the liver and lungs [5, 6]. Thus, although CRC is associated with poor lifestyle choices, genetic mutations play a crucial role in morbidity and mortality [7]. However, since CRC, like all cancers, is a collective term for a variety of different cancer entities, there is no one-size-fits-all solution to treatment.

The *PIK3CA* gene encodes for the catalytic subunit p110α of class IA phosphatidylinositol 3-kinases (PI3K) [8]. In vivo and in vitro research has shown that mutations within this gene are associated with poor prognosis for cancer patients and resistance to standard treatments such as chemotherapy and monoclonal antibody therapy [9–18]. Mutations in the PI3K/Akt/mTOR (mechanistic target of rapamycin kinase)-pathway are amongst the most common across different types of cancer, including not only CRC but also lung, breast, and prostate cancer [19], which have collectively killed more than three million people worldwide in 2020 [1]. This signaling pathway is crucial in cell cycle regulation making it a promising target for anti-cancer drug therapy [20].

Aspirin is one of the most commonly used drugs worldwide. It is widely available and affordable for a large portion of the world’s population as an over-the-counter-drug. There are studies implying that regular intake of low-dose aspirin (acetylsalicylic acid, ASA; ≤ 150 mg per day) may have a chemopreventative and even curative effects on CRC [21–27]. Nonetheless, many health experts advise against prophylactic intake of aspirin due to possible side effects, such as gastrointestinal ulceration and bleeding as well as hemorrhagic stroke [28, 29]. In recent years, there has been increasing evidence that, in the context of CRC, solely patients with *PIK3CA* mutations benefit from the use of aspirin. Therefore, the *PIK3CA* mutation status has been suggested as a predictor for the efficacy of aspirin treatment in CRC [30–34]. However, the mechanisms behind this clinical phenomenon are yet unclear. In the following, we will discuss several signal transduction pathways whose deregulation may be mechanistically related to the effects of *PIK3CA* mutations in cancer development and progression, as well as to particularities of aspirin action in affected individuals.

## The PI3K/Akt/mTOR-Pathway

**Fig. 1:**
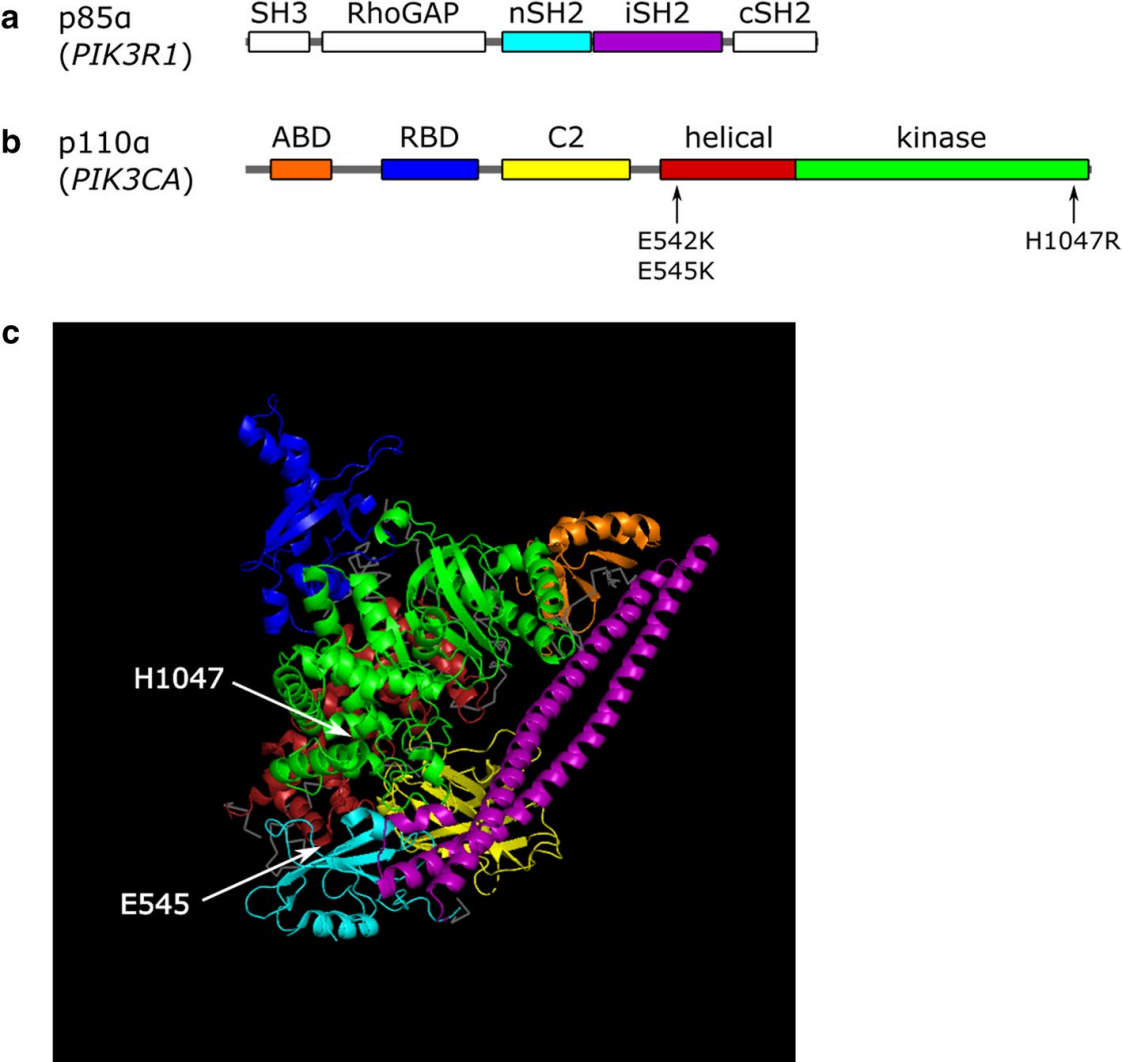
Structure of PI3Kα. **a** Regulatory subunit p85α (NM_181523.3) which is encoded by the *PIK3R1* gene consists of five domains: a Src homology 3 (SH3) domain, a Rho-GTPase-activating protein (RhoGAP) domain, an N-terminal SH2 domain (nSH2; cyan), an inter-SH2 domain (iSH2; purple) and a C-terminal SH2 domain (cSH2). The niSH2 domain is depicted in image c, the crystal structures of the white domains are not yet available. **b** Catalytic subunit p110α (NM_181523.3), encoded by *PIK3CA,* also consists of five domains: an adaptor binding domain (ABD; blue), a Ras-binding domain (RBD; orange), a C2 domain (yellow), a helical domain (red) and a kinase domain (green). The most common *PIK3CA* mutations are E542K and E545K in the helical domain as well as H1047R in the kinase domain. **c** 3D-model of p110α in complex with the niSH2 domain of p85α generated using PyMOL software (4OVU, PDB [41]). The domains are colored according to images **a** and **b**

PI3K are a group of lipid kinases that regulate highly conserved signaling pathways involved in cell proliferation, adhesion, motility, apoptosis, and angiogenesis, thus influencing important hallmarks of cancer [8, 35, 36]. PI3K phosphorylate phosphoinositides at the D3-position of the inositol ring leading to the generation of second messengers. Three classes of PI3K are known [37]. Class I PI3K are heterodimers consisting of a regulatory subunit p85 and a catalytic subunit p110 [38]. The catalytic subunit p110α of class IA PI3K is made up of five domains: an adaptor binding domain (ABD), a Ras (rat sarcoma viral oncogene)-binding domain (RBD), a C2 domain, a helical domain and a kinase domain (Fig. 1a) [8]. The p85α subunit also consists of the following five domains: a Src homology 3 (SH3) domain, a breakpoint-cluster region homology (BH) domain flanked by two proline-rich regions, also known as Rho-GTPase binding (RhoGAP) domain [39], an N-terminal SH2 domain (nSH2), an inter-SH2 (iSH2) domain and a C-terminal SH2 domain (cSH2) (Fig. 1b). The nSH2 domain is responsible for the regulation of p110α and the iSH2 domain is required for tethering (Fig. 1c) [8, 40].

**Fig. 2:**
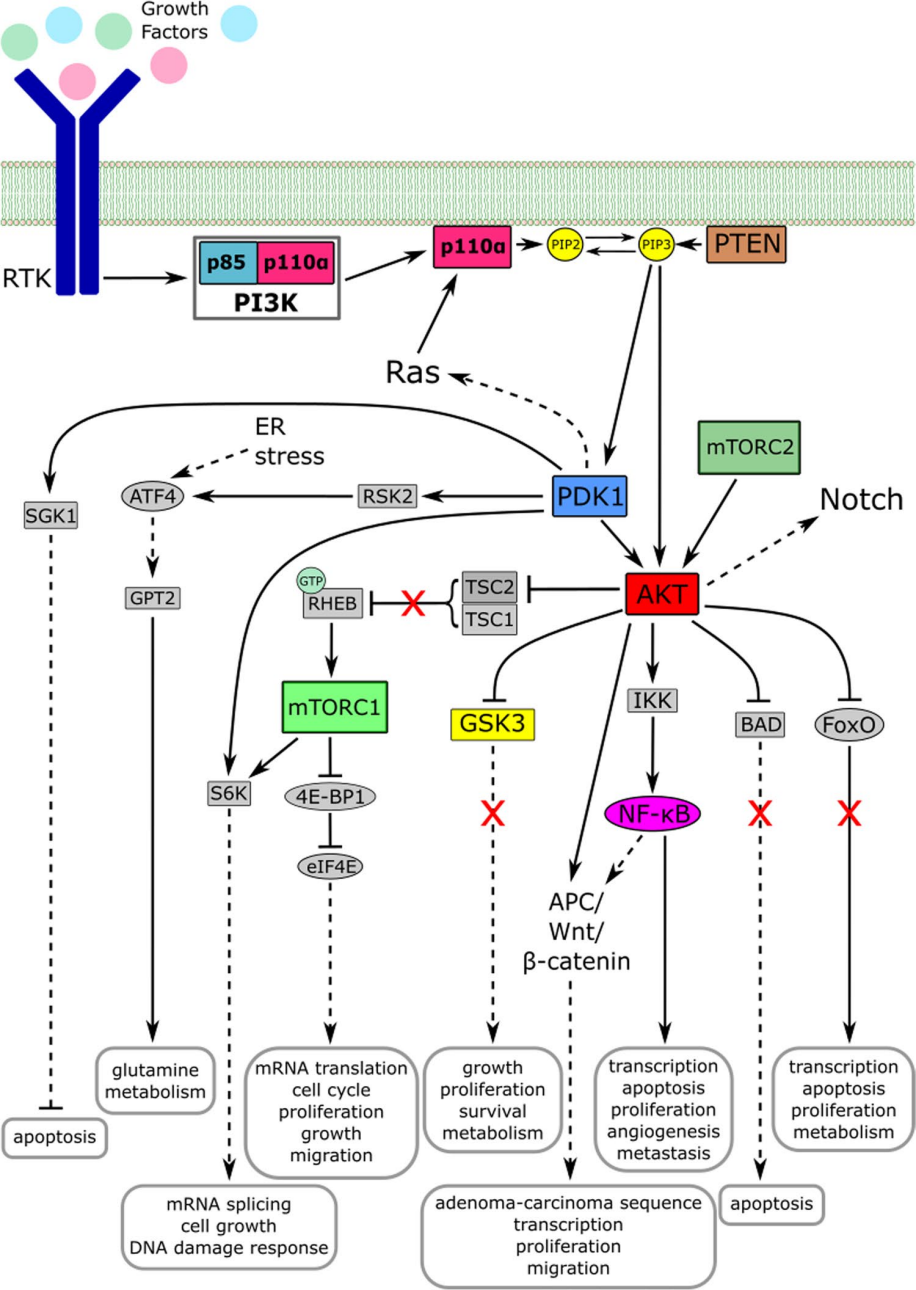
Simplified depiction of the PI3K/Akt/mTOR-pathway. PI3K is activated by RTK or related adaptor proteins. Upon activation, it dissociates and the catalytic subunit p110α is recruited to the cell membrane, a process in which Ras family GTPases assist. At the membrane, it phosphorylates PIP_2_ to the second-messenger PIP_3_, which then activates a complex network of signaling pathways via Akt/PKB and PDK1. The key pathways influencing cell growth, proliferation, and survival, as well as metabolism, gene expression, angiogenesis, and metastasis are displayed. The PI3K/Akt/mTOR-pathway is also connected to other signaling pathways, such as the Ras/Raf/MEK/ERK-, Notch-, and APC/Wnt/β-catenin-pathway. The most important signaling effectors are highlighted in color. Solid lines indicate direct interaction whilst dashed lines indicate indirect interaction downstream. Transcription factors are marked by oval encircling [49, 50, 53, 58–62, 65–72]

Class IA PI3K are mainly activated by growth factor receptor tyrosine kinases (RTK) or associated adaptor proteins [38, 42–44]. Ligand binding of growth factor receptors leads to autophosphorylation of RTK, which in turn leads to binding of the p85 SH2-domain to phospho-YXXM-motifs (pY; X indicating any amino acid) within the RTK. Upon this, the heterodimer dissociates and p110α are released from autoinhibition by p85α, revealing the catalytic site [45–47]. Following dissociation, p110α is recruited to the plasma membrane, a process mediated by GTPases of the Ras family [48]. At the membrane p110α phosphorylates its substrate phosphatidylinositol 4,5-bisphosphate (PIP_2_) to phosphatidylinositol 3,4,5-trisphosphate (PIP_3_) under use of adenosine triphosphate (ATP). The second messenger PIP_3_ in turn recruits adaptor and effector proteins with a pleckstrin homology (PH) domain, including Akt (also known as protein kinase B, PKB) and phosphoinositide-dependent kinase 1 (PDK1), to the cellular membrane. PIP_3_ is later recycled to PIP_2_ by PTEN (phosphatase and tensin homologue, deleted on chromosome 10) [8]. Akt is activated by phosphorylation of T308 and S473 by PDK1 and mTORC2 (mechanistic target of rapamycin, complex 2), respectively [20, 49, 50]. The serine and threonine kinase Akt/PKB is a key player in the regulation of cell survival, proliferation, metabolism, and growth [51–53]. The PI3K/Akt/mTOR pathway is also connected to other pathways such as the Ras/Raf/mitogen-activated protein kinase kinase (MEK)/extracellular signal-regulated kinase (ERK), NF-κB (Nuclear factor κ B), Notch, and APC/Wnt/β-catenin pathways which are also highly involved in carcinogenesis [50, 54–62]. The PI3K pathway as well as the most important connecting pathways is depicted in Fig. 2.

Disturbances in the PI3K/Akt/mTOR pathway play a central role in cancer, the most common deregulations being a loss of PTEN, amplification of the *PIK3CA* or *AKT* gene locus and *PIK3CA* mutations [63–65]. This signaling pathway has, therefore, been suggested as a promising target for anti-tumor agents [20, 66].

## *PIK3CA* mutations in Cancer: results from clinical and translational studies

**Fig. 3:**
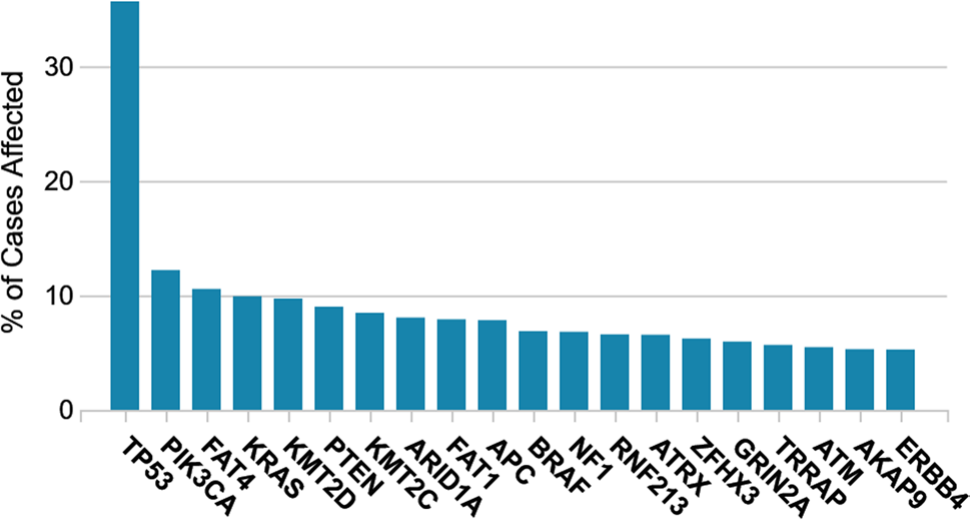
Distribution of the most frequently mutated genes in cancer. According to the US National Cancer Institute *PIK3CA* mutations are the second most common mutation in cancer (12.2% of cases), following *TP53* mutations [19]

The *PIK3CA* gene encodes for the catalytic subunit p110α of class IA PI3K. According to The Cancer Genome Atlas and the US National Cancer Institute, *PIK3CA* is the second most mutated gene across several major cancer types investigated, resulting in a mutational frequency of over 12% (Fig. 3) [19, 73]. Mutation frequency data vary among studies, with the highest values observed for hepatocellular carcinoma (36%) [74], CRC (32%) [75], breast cancer (25–44%) [76–80], head and neck squamous cell cancers (19%) [81], and ovarian cancer (12%) [82].

Three so-called hotspots of mutation have been identified, accounting for more than 75% of all *PIK3CA* sequence alterations [75]: two on exon 9 (E542K and E545K) in the helical domain at the interface of the p85/p110α binding site and one on exon 20 (H1047R) in the kinase domain (see Fig. 1) [40, 83]. These gain-of-function-mutations lead to an increase in PI3K activity and are oncogenic in vivo*.* [84–86] Molecular analyses have shown that the mechanism by which the gain of activity is achieved depends on the position of the mutation [46]. All three hotspot mutations are single-nucleotide substitutions that result in amino acid sequence changes. Mutations of the helical domain (e.g. E545K and E542K) lead to an instability of the nSH2-helical domain inhibitory contact [46, 87, 88]. E545K, for example, induces this by opposite amino acid charge compared to the wildtype causing a conformational change at the nSH2-helical interface [87]. Hence, helical domain-mutated p110α is independent of RTK activation; however, Ras-GTP activation is still necessary. Kinase domain mutations, such as H1047R, however, do not require activation by Ras-GTP, but are dependent on activation via p85 [46, 89]. Kinase domain mutation H1047R also leads to a conformational change, which increases membrane binding of PI3K and, thus, the turnover of the membrane-bound substrate PIP_2_ [88, 90] In vitro experiments have shown that helical and kinase domain mutations act independently of each other and synergistically, if combined [46].

PI3K/Akt signaling plays a major role in the development of CRC and other cancers [91]. *PIK3CA* mutations lead to increased proliferation, reduced apoptosis, and tumor invasion [84]. Yet, the predictive value of *PIK3CA* mutations for clinical outcome is controversial [92]. Many researchers argue in favor of using it as a prognostic marker. For instance, activation of the PI3K/Akt pathway is linked to poor prognosis in CRC [11, 13, 17] and multiple studies report patients with *PIK3CA* mutations to have an overall higher rate of recurrence and metastasis, as well as lower survival rates [11–13, 93]. Moreover, this correlation was not only seen in CRC, but also in other types of cancer, most notably breast cancer [92].

The greatest obstacles in cancer treatment are therapy resistance and tumor recurrence. Common treatments include surgery, pharmacotherapy, and radiation. Standard first-line pharmacological treatments against CRC are chemotherapy regimens like FOLFOX (folinic acid, 5-fluorouracil (5-FU), and oxaliplatin) or FOLFIRI (folinic acid, 5-FU, and irinotecan) in combination with monoclonal antibodies against growth factors or RTK such as VEGF-A (vascular endothelial growth factor A), e.g. bevacizumab, and EGFR (epidermal growth factor receptor), e.g. cetuximab or panitumumab [92]. *PIK3CA* mutations often coincide with therapy resistance [9]. There are multiple reports that *PIK3CA* mutations lead to resistance to targeted treatments against RTK [14, 15, 18]. This was the case in a study conducted by Perrone and colleagues. After sequencing samples derived from patients who did not respond to cetuximab treatment, they found that therapy resistance often coincided with *KRAS* mutation or deregulated PI3K signaling by mutations/loss of *PIK3CA* or *PTEN* [14]. A larger study by Sartore-Bianchi et al. yielded comparable results [18]. After conducting in vitro experiments with *PIK3CA*-mutated CRC cell lines, Jhawer et al. propose *PIK3CA* mutational status as a predictive biomarker regarding the efficacy of anti-EGFR therapy [15]. Patients with *PIK3CA*-mutated CRC have also shown resistance to chemotherapy regimens like FOLFOX or FOLFIRI as well as radiotherapy [16, 94]. Wang et al. found *PIK3CA* mutations to be responsible for the insensitivity of CRC stem cells towards first-line chemotherapy regimens [16]. In light of this evidence, inhibitors of the PI3K/Akt/mTOR-pathway as means of targeted treatment came into focus [65]. Indeed, administration of Akt and mTOR inhibitors in combination with anti-EGFR treatment to *PIK3CA*-mutated cell lines derived from CRC patients has shown promising results, at least in vitro [95]. Morii et al. also found that the Akt inhibitor perifosine can overcome chemoresistance of *PIK3CA*-mutated CRC cell lines to oxaliplatin and 5-FU [96]. As a result of *PIK3CA*-related drug development, the first selective inhibitor of PI3K p110α, alpelisib, was approved by the US Food and Drug Administration (FDA) in 2019 as treatment for *PIK3CA*-mutated hormone receptor positive/human epidermal growth factor receptor 2 negative (HR + /HER2-) breast cancer in combination with the estrogen receptor antagonist fulvestrant in post-menopausal women and men. The approval was based on the SOLAR 1 study, in which progression-free survival at a median follow-up of 20 months nearly doubled (11 vs. 5.7 months) for patients harboring *PIK3CA* mutations receiving the drug compared to the placebo control group. In contrast, patients without *PIK3CA*-mutated cancer did not benefit from alpelisib treatment [97, 98]. In addition, clinical trials have been set up to study whether patients suffering from metastatic colorectal cancer (mCRC) will benefit from alpelisib treatment. For instance, preliminary results from 102 patients enrolled in a combined phase 1b/randomised phase 2 clinical trial (NCT01719380) investigating the safety and efficacy of the BRAF inhibitor encorafenib and the monoclonal EGFR antibody cetuximab with or without alpelisib in patients suffering from advanced BRAF-mutant mCRC indicated that these patients may benefit from therapeutic intervention with alpelisib. Analysis of progression-free survival comparing the triplet to the doublet after 73 events showed a hazard ratio (95% confidence interval) of 0.69 (0.43–1.11; *P* = 0.064) with an overall response rate of 27% (16–41%) and 22% (12–36%), respectively [99]. A 2021 preclinical in vitro trial conducted by Aslam et al. showed that *PIK3CA*-mutated colon carcinoma cell lines are especially sensitive to simultaneous treatment with alpelisib and cyclin-dependent kinase 4 and 6 inhibitor ribociclib compared to wild-type cells [100]. Currently, the ALCAP clinical trial is underway to evaluate the benefits of combined alpelisib and capecitabine, a 5-FU prodrug, treatment in patients with *PIK3CA*-mutated mCRC (NCT04753203) [101]. Also, there is an ongoing study examining the efficacy of PI3K inhibitor MEN1611 in combination with cetuximab, also concerning *PIK3CA*-mutated mCRC (C-PRECISE-01, NCT04495621) [102].

## Pharmacology of aspirin (acetylsalicylic acid)

### History of aspirin

Aspirin (acetylsalicylic acid, ASA) is one of the most widely distributed drugs worldwide. As a non-opioid analgesic, it is widely used to treat pain, fever, and also inflammation. Indeed, for centuries salicylates found in plants, such as the willow or meadowsweet, have been used as remedies against the ailments mentioned above [103, 104]. ASA was first synthesized by Felix Hoffmann and Arthur Eichengrün in 1897 and subsequently distributed by Bayer under the name “Aspirin”. Being the first non-steroidal anti-inflammatory drug (NSAID), it quickly became popular as a “wonder drug”. Its popularity has, albeit, somewhat declined since due to common side-effects, such as gastrointestinal ulceration and bleeding, and the availability of other NSAIDs and acetaminophen (paracetamol) [103]. Although, first intended as a prodrug to avoid the gastric irritation often caused by salicylic acid, the antithrombotic properties of aspirin were discovered in the late 1960s and, thus, widespread use as an antiplatelet agent for the prevention and treatment of thromboembolic complications in the setting of cardiovascular disease has since expanded the prescribing scope even further [103, 105, 106]. Accordingly, the WHO has listed aspirin as an essential medicine since 1977 [105].

### Safety of aspirin

Even though aspirin is considered a relatively safe drug, there are risks associated with its intake, especially if taken long-term [107]. The most common adverse effects of aspirin are an increased risk of bleeding and injuries to the gastroduodenal mucosa [28, 29, 107, 108]. While fatal bleeding incidents are rare, life-threatening complications, such as intracranial and gastrointestinal hemorrhage, do occur and should be considered [28, 29, 109]. Gastrointestinal side-effects are the most frequent; these range from dyspepsia to gastrointestinal bleeding and peptic ulcers [28, 29, 110–112]. Particular caution should be heeded when prescribing long-term aspirin use to the elderly (≥ 75 years), a group that also has an increased risk of developing malignant colorectal tumors [4, 108]. Since most trials regarding the safety of aspirin were conducted in middle-aged patients, risk assessment for the elderly is difficult [28, 108]. The ASPREE trial (Aspirin in Reducing Events in the Elderly), conducted from 2010 to 2014 in the USA and Australia, found that participants (ages 70 and older; ≥ 65 years for Hispanic and black participants in the US) taking 100 mg of aspirin daily actually had higher all-cause mortality than those receiving placebo. Most deaths in the aspirin group were attributed to cancer, mainly of the gastrointestinal tract (including CRC) [113]. Therefore, health experts only recommend prophylactic long-term use of low-dose aspirin (min. 10 years; ≤ 150 mg) for patients aged 50–69 with a life expectancy of at least 10 years and an increased risk of developing CRC or cardiovascular disease [28, 29].

### Pharmacokinetics of aspirin

Following oral ingestion, aspirin is absorbed from the gastrointestinal tract in its unhydrolyzed form by passive diffusion [107, 114, 115]. Aspirin is absorbed from the gastrointestinal tract, with the main areas of absorption being the small intestine and, to a lesser extent, the stomach [115–117]. However, the quantity of non-absorbed aspirin reaching the colon and rectum is unclear, raising the question of whether colorectal cancer or epithelial cells are exposed to aspirin that passes through the colonic lumen or exclusively to aspirin that circulates in the bloodstream after absorption in more proximal portions of the intestine.

After absorption, it is cleaved by esterases into an acetyl moiety and its primary metabolite salicylic acid by cells of the gut mucosa and (primarily) the liver following first-order kinetics [107, 114, 115, 118, 119]. Free acetate yielded by hydrolysis can enter the tricarboxylic acid (TCA) cycle [114]. Plasma levels of unhydrolyzed aspirin rise quickly after absorption and peak after about 15–20 min. Thereafter, they decrease rapidly, while the salicylate levels increase [120]. According to Needs et al. the peroral bioavailability of aspirin is approximately 70%, indicating that about 70% of a perorally administered aspirin dose enters the systemic circulation unhydrolyzed [115]. However, the absorption rate depends on the kind of formulation; aqueous solutions or fast-dissolving formulations lead to quicker absorption from the gastrointestinal tract and higher plasma levels of non-metabolized aspirin [107, 121]. In the blood stream, aspirin, as well as salicylate, is bound to proteins, mainly albumin, and distributed throughout the body [107, 114, 122, 123].

### Mechanism of action of aspirin

Aspirin’s main mechanism of action is the acetylation of proteins, which is entirely non-specific [124, 125]. Examples include the acetylation of human serum albumin, resulting in altered protein function due to conformational changes [124, 126]. Additionally, aspirin has shown to acetylate tumor suppressor protein p53 [19, 124, 127]. There are also accounts of aspirin-acetylating histones, for instance lysine residues such as K56 and K122 on histone H3 [128, 129]. In this context, Wang et al. identified a total of 523 proteins to be targets of aspirin acetylation in HCT116 CRC cells, amongst them mTOR and others affected by the PI3K pathway, such as eIF2 and eIF4A1. In further experiments they demonstrated that aspirin does indeed suppress mTOR function as evidenced by a reduction in phosphorylation (Ser235/236) of the mTORC1 target S6 in HCT116 cells and mouse embryonic fibroblasts [130]. Nevertheless, the causality between specific acetylation of mTOR and mTOR inactivation needs to be verified in future experimental analyses by exchange mutations of the detected acetylated amino acid residues.

The most well-known effect of aspirin, to date, is its influence on the prostanoid system which was discovered by John Vane in 1971 [131]. Prostanoids are a class of lipid mediators that can be divided into prostaglandins and thromboxanes. They derive from the unsaturated fatty acid arachidonic acid which is released from the plasma membrane by phospholipases A_2_ [132, 133]. Cyclooxygenases (COX), also known as prostaglandin G/H synthases (PGHS) or prostaglandin-endoperoxide synthases (PTGS), catalyze the transformation of arachidonic acid into prostaglandin H_2_ (PGH_2_) which is a precursor prostanoid [132–134]. COX exist in two isoforms, COX-1 and COX-2. Commonly, COX-1 is seen as a constitutive form responsible for the maintenance of the gastroduodenal mucosa and tissue homeostasis in general, whereas COX-2 has been identified as an inducible form in most cell types which is mainly expressed in response to inflammation [132], although this static role allocation has been questioned [135]. Aspirin covalently acetylates COX-1 at S530 and COX-2 at S516, thereby irreversibly inhibiting the production of PGH_2_ [107, 136]. The irreversibility of COX inhibition distinguishes aspirin from other “traditional” NSAIDs, such as ibuprofen, which reversibly inhibit the activity of COX-1 and COX-2 through competitive antagonism with arachidonic acid at the active site of the enzymes [107, 137, 138]. The duration of aspirin’s effect is, therefore, also not limited by its half-life but by the turnover rate of the target protein and, in the case of therapeutic use of aspirin as an antiplatelet agent, by the platelet lifespan [107]. Although aspirin has the potential to inhibit both COX isoforms, it is more selective towards COX-1, with IC_50_ values at isolated enzymes of 5 µg/mL (COX-1) and 210 µg/mL (COX-2), respectively [139, 140]. In contrast, most NSAIDs are predominantly nonselective in inhibiting COX-1 and COX-2, although agents such as meloxicam, nimesulide, and diclofenac have been reported to possess a 18- to 29-fold greater potency towards COX-2 in vitro [138, 141]. In addition, selective COX-2 inhibitors act preferentially on COX-2 and, therefore, appear to differ from nonselective COX inhibitors with regard to the spectrum of side effects. As such, they are less likely to cause gastric and duodenal ulceration, whereas they increase the incidence of thromboembolic events and renovascular hypertension, the latter two side effects being most likely due to a reduction in the bioavailability of vascular endothelial (vasculoprotective) prostacyclin with, however, preserved thromboxane A_2_ (TxA_2_)-dependent platelet aggregation and vasoconstriction [142, 143]. Indeed, COX-2-selective inhibitors rofecoxib and valdecoxib have been withdrawn from the market due to an increased risk for the occurrence of cardiovascular events, including myocardial infarction [143].

PGH_2_ formed by COX is then further processed into a variety of prostaglandins and thromboxane by specific synthases [132, 133]. The most relevant being prostaglandin E_2_ (PGE_2_), D_2_, F_2α_, prostacyclin (PGI_2_) and TxA_2_. While both COX isoforms lead to the biosynthesis of prostaglandins, TxA_2_ synthesis predominantly depends on COX-1-derived precursors [132, 133, 144]. This dependence of TxA_2_ formation on COX-1 activity is due to COX isoform-preferential coupling of synthases. COX-1 predominantly interacts with thromboxane A synthase 1 (TBXA1S) and with PGF and cytosolic PGE synthases, whereas COX-2 primarily couples to prostacyclin synthase and microsomal PGE synthases, the latter two being induced by cytokines and tumor promotors [133]. In line with this concept, the COX-2 selective inhibitor celecoxib in doses of up to 800 mg did not inhibit TxA_2_-induced platelet aggregation in humans, while reducing systemic prostacyclin biosynthesis as indicated by a reduction in urinary excretion of the prostacyclin metabolite 2,3-dinor 6-keto-PGF_1α_ in these individuals [142]. Prostanoids act locally and mediate their effects via G-protein coupled receptors (GPCR) [132, 133]. They are ubiquitously expressed throughout the body, where they fulfill a wide variety of functions [134]. For example, PGE_2_ is involved in the response to inflammation and nociception [133, 134]. Moreover, prostaglandins such as PGE_2_ and PGI_2_ contribute to renal blood flow and function and, in the case of PGE_2_, also to gastroduodenal mucosa protection [145]. Prostanoids also play an important role in the regulation of the cardiovascular system, most notably TxA_2_ and PGI_2_. TxA_2_ induces vasoconstriction, platelet aggregation, and endothelial dysfunction, whereas PGI_2_ exerts opposite effects which is why it is considered a protective functional antagonist of TxA_2_ [146].

However, not only formation of the mentioned prostaglandins and thromboxane A_2_ depend on the activity of COX enzymes, but also COX have been shown to be involved, for instance, in dihomoprostaglandin formation from adrenic acid [147] or in the (mostly COX-2-dependent) formation of the F2-isoprostane and thromboxane A_2_ receptor agonist 8-iso-PGF_2α_ (8-iso-prostaglandin F_2α_) [144, 148]. Uninhibited, COX also generate a small amount of the arachidonic acid derivates 11-(R)-hydroxyeicosatetraenoic acid (11-HETE), as well as a racemic mixture of 15-(S)- and (R)-hydroxyeicosatetraenoic acid (15-HETE), the majority being the (S)-enantiomer [137, 149]. Unlike other NSAIDs, aspirin has been reported to qualitatively alter the enzymatic substrate specificity and activity of COX-2. Indeed, aspirin-acetylated COX-2 is involved in the formation of aspirin-triggered specialized proresolving mediators, including aspirin-triggered lipoxins and resolvins, which appear to play a role in the anti-inflammatory and anti-cancer effects of the drug [150, 151]. In this context, acetylation of COX-2 does not completely inhibit its function [137, 149]. Conformational changes due to the acetylation by aspirin lead to a shift in the stereochemistry of the product, resulting in a higher ratio of 15-(R)-HETE formation compared to 15-(S)-HETE (3:1) [137, 149, 152]. Whereas 15-(S)-HETE has been shown to be involved in the process of inflammation, angiogenesis, and the pathogenesis of cancer, especially CRC [153–159], 15-(R)-HETE can be converted to, i.e. 15 epi-lipoxin A_4_ or B_4_ by leukocyte 5-lipoxygenase in a transcellular process during the interaction of COX-2-expressing endothelial or epithelial cells with polymorphonuclear leukocytes [151]. In a similar transcellular fashion, aspirin-induced acetylation of COX-2 initiates the formation of so-called aspirin-triggered resolvins from docosahexaenoic acid (DHA) and eicosapentaenoic acid (EPA) [151]. Resolvins are categorized as either D-series (derived from DHA) or E-series (derived from EPA) and aspirin-triggered epimers have been identified for each family, although compared with the pharmacodynamics of other NSAIDs, a unique activity of aspirin-acetylated COX-2 appears to be its participation in the formation of aspirin-triggered resolvins of the D-series [160]. In this regard, both resolvins and lipoxins promote the resolution of inflammation by stimulating phagocytosis of cellular debris and counteracting the release of pro-inflammatory cytokines without being immunosuppressive [151]. In addition, they have been reported to counteract tumor growth in animal models [161, 162]. Compared with native resolvins, aspirin-triggered forms (R-epimers) appear to resist rapid inactivation by oxidoreductases more effectively and, therefore, have longer half-lives, and as shown, for example, for aspirin-triggered resolvin D1, they appear to have greater efficacy in mediating anti-inflammatory effects, at least in murine models of inflammation [163]. Interestingly, both aspirin-triggered lipoxins and resolvins have been attributed significant anticancer effects in preclinical studies by promoting phagocytosis of apoptotic and necrotic tumor cells by macrophages and counteracting macrophage-induced secretion of pro-inflammatory cytokines [164], suggesting that this pharmacodynamic property of aspirin may be clinically relevant with respect to the anti-inflammatory and anticancer effects of the drug. Nevertheless, it is unclear to what extent these aspirin-triggered lipid mediators play a role in the aspirin sensitivity of *PIK3CA*-mutated CRC. Yet, it is conceivable that activating *PIK3CA* mutations may lead to (i) increased expression of COX-2 in cancer or cancer-associated endothelial cells, (ii) to increased interaction between polymorphonuclear leukocytes and *PIK3CA*-mutated colorectal cancer cells, or (iii) to interaction-induced increased expression of 5-lipoxygenase in polymorphonuclear leukocytes, thereby increasing the efficacy of aspirin-triggered local formation of lipid mediators with potential anticancer activity.

Apart from the acetyl moiety, salicylic acid also interacts with signaling pathways independently, making aspirin one drug with two pharmacologically active compounds [107, 114]. So far, it is not clear if the anti-cancer properties exhibited by aspirin are mediated by acetylation or its salicylate moiety. The mechanism of action of salicylic acid is more complex and, thus, not yet well understood. Salicylate is also known for its anti-inflammatory effect. One possible explanation is its interference with COX-2 expression. There are multiple reports of salicylate inhibiting COX-2 mRNA transcription [165–167]. Xu et al. found that COX-2 mRNA transcription inhibition was mediated by interleukin-1β (IL-1β), phorbol 12-myristate 13-acetate and lipopolysaccharide (LPS) [165]. This is consistent with a 2002 study published by Cieslik and colleagues. They stated that salicylate suppresses both COX-2 as well as inducible nitric oxide synthase (iNOS) expression by reducing the binding CCAAT/Enhancer-binding protein-β to the promoter, which is stimulated by LPS and the cytokine interferon-γ (IFN-γ) [166]. Later, Chae et al. reported that salicylate inhibits phosphorylation of IκBα (NFKB inhibitor alpha) and subsequent degradation mediated by the cytokine TNFα (tumor necrosis factor α) via the ERK1/2-MAPK-pathway. The degradation of IκBα is necessary to activate NF-κB downstream and induce COX-2 expression [167]. According to Wang and Brecher’s 1999 study, salicylate inhibits the expression of iNOS by means of ERK, MAPK, IFN-γ, and TNFα [168]. Kiss et al. also found that salicylate decreases NF-κB activity via inhibition of IκB (IκB protein) [169]. As a downstream target of Akt NF-κB is substantially involved in the oncogenic activity of the PI3K pathway (Fig. 2) [69, 170–172]. According to Hawley et al. salicylic acid also activates metabolic regulator adenosine monophosphate–activated protein kinase (AMPK) [173]. Taken together, these data suggest that the salicylate moiety of aspirin may significantly counteract the *PIK3CA* mutation-triggered overactivity of several relevant effectors of the PIK3 pathway, e.g., by inhibiting NF-κB-dependent signal transduction and by downregulating COX-2 expression, thus possibly contributing to the beneficial effects of aspirin on *PIK3CA*-mutant CRC.

## Aspirin sensitivity of *PIK3CA*-mutated CRC

There is substantial evidence that aspirin is effective in the prevention and treatment of CRC. For example, in a randomized controlled trial involving over 1,000 patients, Baron et al. found that low-dose aspirin (81 mg) had a chemopreventive effect on colorectal adenoma [25]. Also, in a long-term cohort study performed in Sweden over a period of more than 20 years, aspirin use was associated with a 35% reduced risk of CRC [174]. In a meta-analysis that included seven trials each on aspirin therapy after the diagnosis of CRC and seven trials on aspirin use before the diagnosis of CRC, Peiwei Li and colleagues concluded that aspirin is effective for the treatment of CRC after diagnosis but not for primary prevention in people at high risk of developing CRC [27]. Interestingly, an overall survival benefit associated with aspirin use after diagnosis was observed in both colon and rectal cancers, although this survival benefit of aspirin use after diagnosis appeared to be limited to patients with COX-2-positive as well as *PIK3CA*-mutant tumors. In agreement with this study, Bastiaannet et al. also found aspirin to lower the risk of mortality in CRC patients post-diagnosis [175]. Also, in gastrointestinal tract cancers, prolonged patient survival has been observed with low-dose aspirin [176]. Similar observations have been made in non-gastrointestinal malignancies, such as head and neck, breast and lung cancer [177–182]. Thus, these findings together suggest that aspirin may exhibit broad anti-cancerogenic effects which are not limited to CRC.

Different landmark studies suggest that the effect of aspirin on cancer is limited to patients with *PIK3CA*-mutated tumors [30–33]. This was the case in an observational study of over 900 CRC patients published in 2012 by Liao et al. in which aspirin use significantly improved survival in patients with *PIK3CA*-mutant CRC, whereas survival in patients with wild-type *PIK3CA* CRC was not improved [30]. Zumwalt et al. compared the effect of aspirin on various colon carcinoma cell lines and found that aspirin treatment of *PIK3CA-*mutant colon cancer cells leads to a downregulation of cell-cycle-related genes and reduced tumor growth in mouse xenografts [33]. Gu et al. confirmed these findings, observing that aspirin induces apoptosis and leads to G_0_/G_1_ cell-cycle arrest only in colon carcinoma cell lines carrying *PIK3CA*-mutations [32]. Nonetheless, the exact mechanism behind the aspirin sensitivity of *PIK3CA*-mutated tumors remains unclear. In the following, we present the current knowledge on this topic and discuss possible mechanisms by which aspirin may inhibit tumorigenesis related to PI3K signaling.

### COX-2-related aspirin-sensitivity of cancer cells

The most obvious answer would be that this phenomenon is related to inhibition of COX enzymes, as they are by far the best researched targets of aspirin [183]. The COX-2/PGE_2_ signaling axis contributes to most hallmarks of cancer, promoting cancer-associated angiogenesis as well as proliferation, survival, migration, and invasion of cancer cells [184]. Also, COX-2 is induced by inflammation, a process strongly linked to the development of cancer, CRC in particular [132, 185, 186]. Colorectal tumors often display elevated COX-2 expression levels as seen in both patient-derived tissues and animal models [187–189]. Importantly, overexpression of COX-2 is associated with poor survival of CRC patients [190], whereas selective COX-2 inhibition in colon cancer cells has shown to suppress tumor growth and to induce apoptosis as well as cell-cycle arrest [191, 192]. With regard to the clinical relevance of COX-2 in the pathogenesis of CRC, Veettil and colleagues published a systematic review in 2019 concluding that the benefit of CRC chemoprevention with COX-2-inhibitor celecoxib outweighed the risk of cardiovascular side effects after reviewing three randomized controlled trials and three post-trial studies which compared the incidence of recurrence of colorectal adenomas in patients given celecoxib at varying doses or placebo [193]. In line with these findings, Valverde et al. found that combined celecoxib and cetuximab treatment inhibited growth and induced apoptosis in non-KRAS-mutated CRC cells in vitro and in mouse xenografts via impairment of the EGFR/RAS/β-catenin/FOXM1 signaling axis [194]. Also, other NSAIDs like sulindac have proven to induce apoptosis in colon cancer cells, thereby pointing to a class effect of COX inhibitors in CRC treatment or prevention that may be attributable to the ability of these drugs to inhibit COX-2 function [188, 195]. There is evidence from non-CRC cancers indicating that COX-2 signaling is related to PI3K signaling. For instance, Uddin et al. observed that COX-2 inhibition via aspirin, a selective COX-2 inhibitor (NS398), and gene silencing by siRNA led to a reduction of Akt phosphorylation, which resulted in cell cycle inhibition and apoptosis of ovarian cancer xenografts in mice [196]. Further, in a study published by Tury and colleagues, the COX-2-selective inhibitor celecoxib was effective against *PIK3CA*-mutated patient-derived breast cancer xenografts, but not against *PIK3CA*-wildtype xenografts [197]. In CRC, Liao et al. found aspirin was most effective on tumors with both *PIK3CA* mutation and COX-2 expression, but the sample size in this study was too small to draw reliable conclusions [30]. Indeed, PI3K has been identified as a downstream target of COX-2/PGE_2_ but may also be involved in the regulation of COX-2 expression in the context of a positive feedback loop [198–205]. In this regard, PGE_2_-driven EP4 activation and a subsequent EP4-related activation of the GRK/β-arrestin/Src/PI3K/GSK3 pathway has been proposed, leading, for example, to nuclear translocation of β-catenin and β-catenin-dependent gene expression [206]. Moreover, an EP4-selective agonist activated the PI3K/ERK pathway in colon carcinoma cells possibly via EGFR transactivation and thereby rescued proliferation suppressed by indomethacin or COX-2 inhibitors [203]. In this context, PGE_2_ was shown to induce EGFR transactivation and subsequent PI3K signaling via EP4, β-arrestin and c-Src in colon cancer cells [202] In addition, PGE_2_ via EP2 and EP4 has also shown to modify the activity of other growth factor receptors, such as insulin-like growth factor 1 receptor (IGFR) [207]. Interestingly, COX-2 mRNA expression and PGE_2_ synthesis may also be regulated by PI3K via insulin-like growth factors (IGF) 1 and 2, as seen in in vitro experiments with Caco-2 colon carcinoma cells. Here, both the inhibition of PI3K and antagonism of the receptor suppressed COX-2 mRNA transcription [200]. Interestingly, and as addressed in the section on aspirin pharmacodynamics, aspirin can alter the enzymatic activity of COX-2 through acetylation such that it generates precursors of aspirin-triggered forms (R-epimers) of lipoxins and resolvins that arise from these precursor molecules in transcellular processes of COX-2-expressing epithelial or cancer cells and polymorphonuclear leukocytes [151]. These lipid mediators have been shown to elicit both anti-inflammatory and anti-cancer effects in preclinical models, the latter presumably by stimulating phagocytosis of tumor cell debris by macrophages and by inhibiting macrophage-induced inflammation [151]. Although the exact background of the generation of aspirin-triggered lipoxins and resolvins in *PIK3CA*-mutated and COX-2-overexpressing CRC remains unclear to date, it can, therefore, be postulated that aspirin mediates enhanced generation of these lipid mediators in the presence of increased COX-2 expression and activity, which in turn may contribute to the antitumor effects of the drug.

However, it must also be noted that there are clinical data that contradict the relevance of COX-2 as an important target of aspirin in *PIK3CA*-mutated CRC. For instance, in a study by Gray et al., aspirin intake led to increased patient survival in colon cancers with increased COX-2 expression, but the *PIK3CA* mutational status determined was not related to this effect [208]. Moreover and interestingly, in a trial by Enric Domingo and colleagues published in 2013, patients with *PIK3CA*-mutated CRC benefited from aspirin, albeit no improvement was seen in the patients being treated with the COX-2-inhibitor rofecoxib [34]. In this context, it must be noted that in most studies *PIK3CA* mutations were not differentiated as to whether they were located in the helical or in the kinase domain. This could explain in part contradictory study outcomes. For example, as described above, the COX-2/PGE_2_/EP4 signaling axis is able to increase PI3K activity via RTK (trans)activation, an effect that is unlikely to have a relevant impact on downstream signaling events with *PIK3CA* mutations in the helical domain because of uncoupling of these mutants from RTK signaling. Off note, this is also the reason why *PIK3CA* mutations in the helical domain are discussed as a cause of resistance to, for example, EGFR inhibitor therapy and are being investigated in clinical trials (C-PRECISE-01, NCT04495621) as a target in such CRC entities [102]. Nonetheless, aspirin could also elicit anti-tumor effects in these cancer entities via downregulation of COX-2 mRNA expression (potentially via the salicylate moiety of the drug) and a shift in COX-2 enzymatic activity (acetylation by aspirin). Thus, the results presented in this section collectively suggest that COX-2 is a relevant target for aspirin sensitivity of *PIK3CA*-mutated CRC, as both *PIK3CA* activity and COX-2 expression and activity may be amplified as part of a positive feedback loop. Contributing to the clinical phenomenon of aspirin sensitivity of *PIK3CA*-mutated CRC could also be increased formation of aspirin-triggered lipoxins and resolvins in COX-2-overexpressing tumors, which may also provide a promising explanation for different clinical outcomes of aspirin- and selective COX-2 inhibitor-treated patients suffering from *PIK3CA*-mutated CRC. However, given the conflicting clinical and experimental results in this context, further research is needed to clarify the exact mechanistic interconnections.

### COX-1- and TXA_2_-related mechanisms

Aspirin is an NSAID with clinically relevant preference for COX-1 and it has the potential to inhibit COX-2 at higher doses [139, 140, 183]. In this context, it seems plausible that the effect of aspirin on *PIK3CA*-mutated CRC is mediated preferentially by COX-1 or TxA_2_, the latter being a prostanoid whose generation depends predominantly on COX-1-mediated PGH_2_ biosynthesis [132, 133]. Angiogenesis, as well as platelet aggregation play an important role in tumor growth and metastasis, both are influenced by TxA_2_ [209–213]. Thus, these processes could be inhibited by aspirin’s anti-platelet properties and in particular through a reduction in platelet TxA_2_ synthesis, which appears to be essential to prepare metastatic intravascular niches at least in preclinical cancer models in vivo [214]. The inhibition of platelet-activated metastasis by aspirin could be mediated by inhibition of Akt, as suggested by a preclinical in vivo study on breast cancer cells [215]. Here, platelets were shown to promote metastasis of these cells through activation of the Akt signaling pathway and aspirin treatment of platelets resulted in inhibition of Akt and subsequent inhibition of pro-metastatic IL-8 production. Also, Li et al. proposed that PI3K is critical for platelet secretion as they were able to stimulate PI3K-dependent phosphorylation of Akt after activation of the thromboxane A_2_ receptor using the TxA_2_ mimetic U46619 [216]. COX-1 activity (and subsequent TxA_2_ formation) may also be involved in the development of CRC as experiments regarding azoxymethane-induced CRC in rats suggest [217, 218]. In line with these findings, Wu et al. observed that the selective COX-1 inhibitor SC-560 induced cell-cycle arrest and macroautophagy in colon cancer cells [219]. A study by Sakai et al. reported that TxA_2_ levels were upregulated in human colorectal carcinomas and CRC cell lines due to overexpression of TBXA1S, resulting in increased cell proliferation [220]. In addition, COX-1 can ease symptoms of colitis by upregulating β-arrestin via PGE_2_ and PI3K/Akt, indicating that the activity of these signaling effectors upstream may also be influenced by COX-1 in the colonic epithelium [221]. Taken together, COX-1 signaling in cancer and its inducible mechanisms have long been overlooked. Therefore, to fully understand the development of CRC and the beneficial effects of aspirin in *PIK3CA*-mutated CRC in this context, further research in this area is essential. However, based on current knowledge, COX-1 appears to act similarly to COX-2 upstream of PI3K and therefore the effect of aspirin on COX-1 activity is unlikely to explain the particular effects of the drug on *PIK3CA*-mutated CRC.

### NF-κB signaling

Another possibility for the aspirin-sensitivity of *PIK3CA*-mutated CRC could be explained by prostaglandin-independent mechanisms. The NF-κB pathway is involved in the response to inflammation and infection, but also cancer development. A study by Martha Slattery and colleagues in 2018 showed that the NF-κB signaling pathway is dysregulated in CRC, as are p53 and Wnt/β-catenin signaling [172, 222]. NF-κB and β-catenin are involved in angiogenesis and metastasis in CRC [170]. Interestingly, both aspirin and salicylate were identified as specific inhibitors of IKK-β (IκB kinase-beta) activity in vitro and in vivo, an effect that depended on direct binding of the drugs to IKK-β to interfere with ATP binding [223]. Indeed, Jinbo Fu and colleagues demonstrated that aspirin suppressed chemoresistance to 5-FU in 5-FU-resistant CRC cell lines SW620 and SW480 by abrogating 5-FU-induced NF-κB activation, thereby enhancing the antitumor activity of 5-FU [224]. Also, salicylate treatment has been suggested to reduce chemotherapy resistance by inhibiting NF-κB activity [225] and it may inhibit Mucin-1 (MUC1)-mediated tumor migration and invasion by inhibiting Akt [226]. Thus, these results suggest that aspirin-mediated inhibition of tumor pathogenesis may also involve modulation of atypical signaling pathways not previously associated with aspirin pharmacodynamics. NF-κB signaling is not only related to PI3K/Akt, but also the Wnt/β-catenin pathway, and their connection is necessary to fully understand PI3K signaling in CRC and aspirin sensitivity of *PIK3CA*-mutant CRC entities.

### Wnt and β-catenin-dependent signaling

The canonical Wnt/β-catenin pathway is one of the most crucial pathways in the development of CRC [227, 228]. It drives cell cycle progression and maintains the undifferentiated state of intestinal stem cells [227]. Pathway activation starts with a glycoprotein of the Wnt family extracellularly binding to the N-terminal domain of a G-protein coupled receptor of the Frizzled family. This activates Dishevelled (DVL), which is then translocated to the plasma membrane. In turn, DVL recruits the destruction complex consisting of APC, glycogen synthase kinase (GSK) 3β, Axin and casein kinase 1, inactivating it by removal from the cytosol [229]. In the cytosol, the destruction complex marks the protein β-catenin for ubiquitination and subsequent degradation by the proteasome [228]. However, unhindered by the complex, β-catenin relocates to the nucleus where it enables the transcription of various proteins responsible for cell differentiation, proliferation, and migration, for example, the proto-oncogene *c-myc* [230]. Wnt/β-catenin signaling is important for the homeostasis of the intestinal epithelium [231]. In complex with E-cadherin, β-catenin forms cell–cell junctions regulating epithelial cell adhesion and polarity. Deregulation may lead to epithelial-mesenchymal transition (EMT) [232]. EMT refers to the process of transition of epithelial cells into cells with mesenchymal properties, in which cells lose cell polarity and cell–cell adhesion to adopt a migratory and invasive phenotype, often considered the first step towards metastasis [233].

It is believed that the majority of CRCs arise via the adenoma carcinoma-sequence, a series of mutational events leading to the gradual transformation of benign polyps into malignant carcinomas [5, 234]. Alterations in the Wnt/β-catenin pathway, especially APC mutations, play a central role in this process [5, 235]. The Wnt/β-catenin and PI3K pathways are closely connected. One example is the connection via GSK3β, a substrate of Akt and member of the β-catenin destruction complex [58, 235]. It is phosphorylated by Akt at S9 leading to its inactivation [236]. Albeit, there is no evidence that phosphorylation of GSK3β by Akt directly influences the activity of the Wnt pathway [237, 238]. Further, Zeng et al. could show that tuberous sclerosis (TSC) 2 deletion and subsequent mTORC1 activation inhibits Wnt signaling by regulating Frizzled protein levels in a DVL-dependent way [239]. This is an interesting finding in the context of *PIK3CA*-mutated CRC because mTORC1 is a downstream target of PI3K-Akt by inhibiting TSC1/2 (see Fig. 2) and because there is evidence for aspirin-mediated direct acetylation and inactivation of mTOR and the mTORC1 complex [130, 240]. Also, a 2007 publication by Fang et al. suggests that Akt directly phosphorylates β-catenin at S552 resulting in dissociation of cell junctions and accumulation of β-catenin in the cytosol and the nucleus of human epidermoid carcinoma cells [241]. In accordance, Steffen Ormanns and colleagues later found that β-catenin transcriptional activity depended on PI3K activity, presumably due to Akt phosphorylation. In this context, PI3K inhibition did not affect the subcellular localization of β-catenin but impaired the binding of β-catenin to Wnt target gene promoters and decreased the expression of Wnt target genes [242]. Apart from this, it also should be noted that both pathways are also connected to NF-κB signaling (see Fig. 2 and previous section) [69, 243, 244].

Interestingly, there is evidence that sensitivity of cancer cells to aspirin is mediated by Wnt and β-catenin signaling. In an in vitro approach to investigate the influence of aspirin on Wnt/β-catenin signaling in CRC, Bos and colleagues demonstrated that aspirin treatment induced a decreased expression of Wnt target genes and increased phosphorylation of β-catenin in DLD-1 and SW480 colon carcinoma cell lines. Phosphorylation of β-catenin most likely led to its ubiquitination, as cytosolic β-catenin subsequently decreased. They, therefore, assumed that this effect is mediated by protein phosphatase 2A (PP2A), a negative regulator of Akt [245, 246]. PP2A is critically involved in cell–cell adhesion and EMT by stabilizing the β-catenin/E-cadherin complex. Interestingly, the findings of Bos et al. are consistent with two studies published in recent years. In 2019 Jin and Wuo observed that aspirin inhibits Wnt-mediated EMT in SW480 colon carcinoma cells [247]. In addition, a paper published in 2021 by Dunbar et al. showed that aspirin could reverse a Wnt-induced stem-like phenotype in intestinal organoids derived from mouse and human organoids lacking APC. In addition, motility and invasion of colon cancer cells were reduced, effects mediated via the induction of Dickkopf-1, a Wnt-antagonist, through aspirin treatment [248]. Taken together, these data suggest that beneficial effects of aspirin on CRC pathogenesis may be mediated, at least in part, by inhibition of Wnt/β-catenin signaling. However, it is unclear to what extent the inhibitory effects of aspirin might be of particular relevance in *PIK3CA*-mutated CRC in this context. Additional future studies are therefore needed to elucidate this issue.

### Cancer stem cells

As discussed in the previous section, Wnt/β-catenin signaling is among the most important factors in CRC carcinogenesis. In addition, it plays an important role in intestinal homeostasis and cancer stem cell (CSC) development, which will be discussed in the following.

The epithelium of the intestine is continually replenished with a turnover rate of approximately 3–4 days [249]. New cells derive from so-called crypts of Lieberkühn which line the intestinal wall [249–251]. These crypts harbor pluripotent intestinal stem cells (ISCs) responsible for renewal of the intestinal mucosa and its homeostasis [249, 251], a process highly regulated by the Wnt/β-catenin pathway [231]. It is conceivable that colorectal CSCs derive from ISCs in a series of mutational events leading to a loss of proliferative control mechanisms [251, 252]. Importantly, CSCs could then represent key players in metastasis, therapy resistance, and CRC recurrence [252, 253]. Most cancer therapies, such as radiation and chemotherapeutic drugs, target replication mechanisms of highly proliferating cells, such as cancer cells [252, 253]. CSCs are usually in a quiescent state, thus, evading those therapies [252, 253]. Even after seemingly successful chemo- or radiotherapy, surviving CSCs may lead to tumor recurrence and metastasis [252, 253]. Specifically targeting CSCs is difficult and the objective of current research. The PI3K/Akt/mTOR and related pathways, such as the Notch and Wnt/β-catenin pathways, have come into focus of this research [253]. Chen et al. observed that the dual PI3K/mTOR inhibitor BEZ235 suppresses the proliferation of CSCs [254]. Wang et al. also linked PI3K/Akt signaling to stem cell-like properties and 5-FU resistance in CRC [255]. They suggested that the observed effect is mediated by metastasis‑associated colon cancer 1 (MACC1), which has also been proposed as a biomarker predicting metastasis, poor prognosis, and therapy resistance [255, 256]. Similar observations have also been made in other cancers: in an in vitro model of therapy resistant ovarian cancer, Thakur and Ray found that NF-κB activates TNFα and PIK3CA via a feedback loop leading to the maintenance of stem cell-like characteristics [257]. According to Zhou et al., activation of the PI3K/Akt/mTOR pathway is responsible for stemness in gastric carcinoma cells [258]. In this context, several studies confirm that NANOG, a transcription factor essential for the maintenance of embryonic and CSCs, is regulated by the PI3K/Akt pathway [255, 259–262]. In CRC, high levels of NANOG are associated with vascularization and more aggressive behavior in general [263, 264]. Indeed, it is possible that CSCs are the target of aspirin’s anti-cancer properties. Chen et al. reported that aspirin (but no other NSAIDs) directly interacts with p300 in the nucleus, promotes H3K9 acetylation, activates FasL expression, and induces apoptosis in patient-derived colorectal CSCs, whereas these effects of aspirin were not observed in non-CSCs most likely because of H3K9 hypermethylation [265]. Interestingly, salicylate and its derivates have also shown to interfere with p300 [266, 267]. Previously, p300 had been reported to be a target of Akt phosphorylation [268]. Wang and colleagues demonstrated that aspirin reduced colorectal xenograft tumor growth in nude mice, an effect that was associated with a reduction in stemness-related transcription factors, such as c-Myc, OCT4, and NANOG. Interestingly, suppression of NANOG was also able to block the anti-tumorigenic effect of aspirin, suggesting that NANOG is an important downstream target of aspirin action in CRC [269]. In esophageal squamous cell carcinoma, aspirin was able to overcome cisplatin resistance in CSCs by inhibiting the phosphorylation of Akt, suggesting an involvement of PI3K. Aspirin most likely acetylated histones leading to altered gene expression, in this case remodeling of chromatin in the region encoding the pro-apoptotic Bcl-2-like protein 11 (BIM) [270]. Both Khoo and Saha suggest that aspirin may overcome chemotherapy resistance in preclinical models of CSCs via interleukin 6 (IL6), a pro-inflammatory cytokine which acts downstream of NF-κB and is regulated by COX-dependent as well as COX-independent mechanisms [271, 272]. Khoo et al. believe the effect to be due to COX-2 inhibition, whereas Saha et al. speculate aspirin to interfere with the IL6-NF-κB-feedback loop [271, 272]. Taken together, the effect of aspirin on CSCs and the involvement of PI3K in their regulation may provide an explanation for how aspirin treatment can overcome treatment resistance and counteract metastasis in *PIK3CA*-mutated CRC. Nevertheless, studies on the influence of aspirin or its salicylate moiety on CSCs carrying activating somatic mutations of *PIK3CA* are lacking. This important and interesting field of research is therefore still in its infancy and further research on this topic is to be expected in the near future.

### Noncoding RNA

Even though a relevant portion of the genome is transcribed, only 1–2% encode for protein and are translated by the ribosome. Nevertheless, for decades, non-coding RNA (ncRNA) was mainly considered as "transcriptional junk" with no biologically relevant function [273, 274]. With accumulating evidence of involvement of ncRNAs in signal transduction, this perception has rapidly changed [275]. The types of ncRNA to be focused on in this review are microRNA (miRNA), long noncoding RNA (lncRNA), and circular RNA (circRNA). These regulatory RNAs have been observed to play a role in the development of CRC and have, therefore, been proposed as therapeutic targets and diagnostic markers [275–278]. Although it has been proven that altered ncRNA expression is a central element in the formation of cancerous entities, the underlying regulatory mechanisms are complex and not well explored [275, 277, 279]. Indeed, according to Anastasiadou et al.*,* ncRNAs act as parts of vast and intricate networks [275].

miRNAs are short noncoding RNA strands with an average length of 22 bases, deriving mainly from intronic sequences [280]. Their most prominent function is post-transcriptional gene regulation by base-pairing to the 3’ untranslated region (3’UTR) of messenger RNAs (mRNAs), thus, hindering translation or inducing degradation of their target mRNAs [281]. Most miRNAs can bind to multiple mRNAs, also their expression and actions may differ in a cell-type specific way [280]. In the context of cancer pathogenesis, miRNA expression has been shown to play a crucial role in the regulation of cell cycle progression, growth, and metabolism, and, thereby, carcinogenesis. For instance, deregulated miRNAs can promote tumorigenesis by both inhibiting the transcription of tumor suppressor genes as well as failing to do so in the case of oncogenes [282].

LncRNAs are defined as transcripts with a length of > 200 nucleotides [282]. Apart from not being translated, lncRNAs are believed to be processed in the same manner as mRNAs, including transcription by RNA polymerase II as well as post-transcriptional modification, such as 5’ capping, polyadenylation, and splicing [283]. LncRNAs interact with DNA, mRNA, miRNA, and proteins in a multitude of ways. For instance, they can regulate gene expression by blocking access of transcription factors, polymerases, or miRNA to DNA or RNA, or they can also act in the opposite manner, by guiding transcription factors towards DNA or by modifying chromatin remodeling and accessibility [284].

CircRNAs, as the name indicates, are not linear strands of nucleotides but closed loops without 5’ or 3’ ends [285]. CircRNA is less explored than miRNA and lncRNA, most of what is known has been discovered within the past decade [286]. It has been suggested that circRNA descends from protein-coding regions of the genome and is generated from pre-mRNA in a process called “backsplicing” [285] and some circRNAs may even be translated [287]. So far, it has been discovered that circRNA can act as a molecular sponge for miRNA by presenting complementary binding sites akin to those of mRNA targets [288].

There is proven crosstalk between the PI3K pathway and ncRNA networks [289]. For example, the *CRNDE* lncRNA transcript is activated via the PI3K/Akt/mTOR and Raf/MAPK pathway by insulin/IGF in colorectal carcinoma cells, resulting in altered metabolism and the induction of the Warburg effect, which describes metabolic changes by which cancer cells switch to anaerobic glycolysis even in the presence of oxygen and fully functioning mitochondria [290]. A paper by Khan and Law further reported that the lncRNA *RAMS-11* is significantly overexpressed in various CRC cell lines, a phenomenon that correlates with increased proliferation and metastasis. Moroever, downregulation of *RAMS-11* resulted in increased apoptosis by inhibition of Akt/mTOR signaling via AMPK [291]. Multiple studies associate the expression of oncogenic lncRNA *HOTAIR* with PI3K signaling [290, 292, 293]. *HOTAIR* may act as a molecular sponge for miRNAs such as miR-34a and mir-206, which are known to interact with the PI3K pathway [294, 295]. In a similar fashion, miR-34a also binds to lncRNA *SNHG7* enabling translation of *GALNT7* [296]. Li and colleagues were able to show that this competition leads to an increase in PI3K/Akt/mTOR activity and related cell proliferation in CRC cells [296]. MiR-206 inhibits Akt and its downstream target GSK3β via c-Met, resulting in reduced proliferation, migration, and invasion of CRC cells [297]. Overexpression of miR-590-3p desensitizes colon carcinoma cells to radiation by promoting PI3K and Akt phosphorylation [298]. Most notably, El-Daly et al. discovered that miR-370 acts as a tumor suppressor by inhibiting *PIK3CA* and *EGFR* mRNA expression through 3’UTR base-pairing in an in vitro and in vivo model of CRC [299]. CircRNAs have shown to influence PI3K/Akt signaling, as well. For instance, circ-0001313 is overexpressed in CRC cells where it induces cell proliferation and inhibition of apoptosis by sponging *AKT2* mRNA-directed miR-510-5p [300]. Circ_0008285 acts as a tumor suppressor by enabling *PTEN* translation, although, however, it appears to be downregulated in CRC cells [301]. Just recently, Chong and colleagues demonstrated that downregulation of circLHFPL2 most likely promotes sustained activation of the PI3K/Akt pathway by acting as a sponge for miR-556-5p and miR-1322 in CRC cells to modulate PTEN expression [302]. These results demonstrate that there are numerous reciprocal interactions between PI3K or PI3K-dependent signal transduction and ncRNA which have been shown to influence the phenotype of CRC entities. However, systematic studies on the impact of activating *PIK3CA* mutations on the expression of ncRNAs in CRC remain surprisingly scarce.

Results from experimental studies indicate that aspirin exerts anti-carcinogenic properties via ncRNA. Examples include a study by Lan et al.*, who* found aspirin to negatively regulate miR-21 expression through inhibition of TCF4, a transcription factor of the Wnt/β-catenin signaling pathway, in patient-derived colon cancer epithelium [303]. Further, Guo et al. could show the involvement of aspirin in the transcription of lncRNA *OLA1P2* in cultured cancer cell lines as well as mouse xenografts*.* Aspirin, they observed, enabled expression of the transcription factor FOXD3 via demethylation of the *FOXD3* promotor, leading to transcription of *OLA1P2*. In turn, *OLA1P2* blocked the nuclear import of phosphorylated STAT3 [304]. STAT3 is a phosphorylation target of mTOR [305]. In a 2021 study, aspirin treatment of colon carcinoma cell lines lead to inhibition of lncRNAs *NEAT1* and LOC152578 resulting in reduced cell growth and metastasis [306]. Although ncRNAs have now come into focus with regard to aspirin-mediated inhibitory effects on CRC pathogenesis, systematic studies on the role of ncRNAs in aspirin sensitivity of PIK3CA-mutated CRC are scarce to date. Nevertheless, ncRNA may play a significant role in mediating the aspirin-induced effects associated with *PIK3CA*-mutated CRC. Therefore, it is expected that future research projects will soon shed light on this hitherto poorly studied issue.

### Gut microbiota

The gastrointestinal microbiome plays an important role in inflammatory processes and the regulation of gut homeostasis, closely communicating with ISCs [307, 308]. Gut microbiota are believed to be involved in carcinogenesis [309]. While some infectious pathogens may drive cancer development, an imbalance in the microbial composition is often found in CRC patients compared to healthy controls [309–311]. *Escherichia coli*, *Streptococcus gallolyticus/bovis, Bacteroides fragilis* and *Fusobacterium nucleatum,* amongst others, have been linked to the development of CRC*.* [310, 312–316] In addition a pathogenic role of *Enterococcus faecalis* in this regard has been discussed but remains controversial [317]. One Factor which may contribute to dysbiosis is nutrition, especially a diet high in fat and low in fiber [309, 310, 313, 318]. There is evidence that the intake of aspirin affects the gut microbiome [319, 320]. In an observational study with healthy volunteers, Rogers and Aronoff noticed that the profile of the gut microbiome of NSAID users differed from that of non-users. In this study, the operational taxonomic units of bacterial species *Prevotella*, *Bacteroides*, *Barnesiella*, and the *Ruminococcaceae* family distinguished aspirin users from those abstaining from any kind of medication [320]. This is consistent with a later study by Prizment et al. who measured the relative abundance of multiple gut bacterial taxa before and after a six week treatment with 325 mg aspirin or placebo. They found relative increases in *Akkermansia, Prevotella,* and *Ruminococcaceae* as well as decreases in *Parabacteroides*, *Bacteroides,* and *Dorea* in the aspirin group compared to the placebo group [321]. In this context, the presence of *Prevotella* and *Ruminococcaceae* have been shown to negatively correlate with CRC, whilst the latter three have been positively correlated with the disease [322, 323]. In the case of *Akkermansia* there is conflicting evidence whether it is considered a beneficial microbe, especially with regard to inflammation [324, 325]. Zhao and colleagues observed aspirin treatment to induce a shift in microbiomal composition. Bacterial genera considered beneficial, such as *Bifidobacterium* and *Lactobacillus,* were enriched, while bacteria associated with CRC, like *Alistipes finegoldii* and *B. fragilis*, were reduced. In addition, they confirmed an inverse effect of aspirin treatment on tumor size and number in mouse xenografts, albeit only in animals treated with antibiotics. Importantly, some microbes, especially *Lysinibacillus sphaericus*, were found to degrade aspirin resulting in lower plasma levels [319]. Caitlin Brennan et al. found that aspirin and its metabolite salicylic acid alter the expression of several genes in the *F. nucleatum* strain Fn7-1 in culture, including genes encoding for chaperones, proteins related to the Wnt/β-catenin pathway, and ptgs2, the gene encoding for COX-2. At higher concentrations, aspirin also inhibited growth of the mentioned *F. nucleatum* strain Fn7-1. Further, they observed that aspirin reduced *F. nucleatum*-related growth of colorectal adenomas in mice. *F. nucleatum* strains isolated from human CRC tissues yielded consistent results. Additionally, aspirin treatment asserted significant antibacterial effects on *B. fragilis* and *E. coli* strains similar to those seen with *F. nucleatum*, although somewhat milder [326]. Interestingly, Han et al. report in a pre-print that infection of Caco-2 and HT-29 CRC cells lines with *F. nucleatum* leads to resistance against cetuximab in vitro and in mouse xenografts coinciding with an activation of the PI3K/Akt and JAK/STAT3 pathways in infected cells [327]. However, to our knowledge, a link between this and the sensitivity of gut bacteria towards aspirin has not yet been investigated, but it might be worth keeping in mind. Thus, to our knowledge, there is no evidence (but also no counterevidence) to date that aspirin sensitivity of *PIK3CA*-mutated CRC is related to the microbiome. Nevertheless, the colon microbial milieu plays an important role in intestinal epithelial homeostasis and carcinogenesis, so this must be taken into account when elucidating the specific pharmacodynamics of aspirin in *PIK3CA*-mutated CRC in future studies.

### Tumor metabolic reprogramming

Changes in metabolism stand at the heart of tumorigenesis and are now considered a hallmark of cancer [328]. Cancer cells have a high demand for nutrients and energy to enable unhindered growth and proliferation, a need that cannot be met by the mechanisms utilized by “ordinary”, non-malignant cells [329]. Otto Warburg already observed metabolic reprogramming of cancer cells nearly a century ago. The Warburg effect states that tumor cells perform anaerobic glycolysis despite an adequate supply of oxygen [330]. This kind of metabolism might be favorable for highly proliferative cells by directing metabolites towards biomass production instead of generating ATP [331–334]. A heightened need for glutamine is also considered a hallmark of cancer metabolism [329]. In the cell, it can be converted to α-ketoglutarate to enter the TCA cycle and not only serves as an energy source, but also yields carbon and nitrogen for biosynthesis [329, 334, 335]. It is likely that metabolic shifts precede somatic mutations in CRC and promote the development of benign adenomas into carcinomas [334]. As metabolic changes are very common in cancer, they can be used for diagnosis and prognosis of disease. Imaging techniques like positron emission tomography (PET) scans are a frequently used option. CRC and other cancers can be identified through increased glucose metabolism, an indicator of the Warburg effect, by detecting positron emission of the glucose analog [^18^F]fluorodeoxyglucose after injection [336]. In addition, PET scan techniques detecting ^11^C-glutamine are currently being established [337]. Serum glutamine levels may also be used as diagnostic and prognostic marker. For instance, in a retrospective study of 123 newly diagnosed CRC patients Ling and colleagues found decreased glutamine levels to correlate with lower overall and progression-free survival [338].

The PI3K/Akt pathway plays a major role in the regulation of metabolism as well as metabolic reprogramming of cancer cells [61]. Phosphorylation of the glycolysis enzyme hexokinase 2 (HK2) by Akt results in increased proliferation, tumorigenesis, and metastasis of colon cancer mediated by NF-κB and hypoxia inducible factor 1α (HIF-1 α) both in vitro and in vivo in mouse xenograft tumors [339]. PI3K/Akt-mediated HK2 signaling has also shown to inhibit apoptosis in pediatric osteosarcoma [340]. In cervical carcinoma, *PIK3CA* mutations E545K and E542K lead to increased glucose metabolism and cell proliferation via AKT/GSK3β/β-catenin signaling in xenografts and in patient-derived samples [341]. Laboratory experiments as well as clinical trials have shown that *PIK3CA*-mutated CRC cells are especially dependent on glutamine for survival compared to *PIK3CA* wildtype cells [67, 342–344]. This might be related to the fact that the expression of glutamine transporter ASCT2 (also known as solute carrier family 1, member 5 (SLC1A5)) is regulated by PI3K-dependent mTOR signaling [345].

In recent years, glucose and glutamine metabolism have come into focus as potential mediators of aspirin’s effect on cancer. Further, there is substantial evidence that it targets the deregulated metabolism of cancer cells in a PI3K-dependent manner. For example, aspirin has shown to inhibit the enzyme glucose-6-phosphate dehydrogenase (G6PD) responsible for NADH production in the pentose phosphate pathway (PPP) by acetylating lysine residues in HCT116 and HT-29 CRC cells [346, 347]. G6PD is regulated by PI3K/Akt as a downstream target of mTORC1 [348–350]. Chen et al. found that PI3K activity decouples glycolysis and the TCA cycle, while promoting PPP through G6PD activation [347]. Further, aspirin downregulated glutamine and glucose levels as well as inflammation and tumor growth in a study of lung cancer in obese mice, effects that were associated with Akt phosphorylation and GLUT1 expression [351]. Experiments concerning a murine model of ulcerative colitis, a kind of inflammatory bowel disease, yielded that both glutamine and 5-aminosalicylic acid (5-ASA) were able to alleviate symptoms caused by oxidative stress-injury induced through inhibition of the PI3K/Akt signaling pathway [352]. According to a paper published by Hao et al. in 2016 *PIK3CA* mutations lead to an upregulation of glutamate pyruvate transaminase 2 (GPT2) in CRC cells via ATF4 (activating transcription factor 4)/PDK1/RSK2 (ribosomal S6 kinase 2) in an AKT-independent manner ultimately rendering the cells dependent on glutamine [67]. In 2020, Shogen Boku and colleagues could prove that administration of aspirin led to the same effect as glutamine deprivation in *PIK3CA*-mutated CRC cell lines. It did so by two mechanisms: G_1_-arrest was induced via the mTOR-pathway and glutaminolysis enzymes w-ere activated via ATF4 [68]. Taken together, one of the most effective methods of preventing tumor growth appears to be to deprive cancer cells of nutrients. The evidence for the effect of aspirin on deregulated metabolism in *PIK3CA*-mutated cancer is considerable. However, no clear metabolic targets have yet been identified in the aforementioned publications to explain the aspirin sensitivity of *PIK3CA*-mutant CRC, so additional research is needed here to elucidate the precise mechanistic background of this phenomenon.

## Summary and conclusion

A vast number of cancer patients harbor *PIK3CA* mutations. Unfortunately, the prognosis of these patients is dismal in many cases, and the efficacy of standard treatments is often reduced. This leads to an urgent need of alternative treatments for these patients. Genetic profiling and subsequent targeted therapy may be a promising perspective for these individuals. PI3K and Akt inhibitors have shown much potential, aspirin, however, is low in cost and easily available. Nonetheless, the U.S. Preventive Services Task Force is cautious to universally recommend low-dose aspirin (< 100 mg/day) as preventative treatment against CRC due to side-effects, such as gastrointestinal bleeding and hemorrhagic stroke [28].

Moreover and importantly, the balance between harm and benefit is yet unclear. Thus, additional evidence is needed to elucidate the mechanisms behind the preventive and curative effects of aspirin with regard to colorectal carcinoma in order to identify patients who particularly benefit from aspirin therapy.

Although the effect of aspirin on the prostaglandin pathway is one of the best-researched drug mechanisms so far, it is still unclear how aspirin is linked to beneficial effects in individuals carrying somatic *PIK3CA* mutations. Aspirin is hydrolyzed in the body into acetyl and salicylic acid within a short time, making it two pharmacologically active compounds in one drug. The acetylation of molecules is nonspecific and is related to the concentration of aspirin at the site of action, which is particularly high in the gastrointestinal tract after peroral ingestion of the drug, although to our knowledge there are no available pharmacokinetic data on how high the luminal concentration of perorally administered aspirin at the colorectal epithelium really is. However, based on such data, specific retardations of aspirin with dissolution in the colorectal intestinal segments could be developed, allowing higher luminal concentrations of aspirin at the colorectal epithelium and thereby possibly enhancing the anticancer effects, while potentially reducing the known gastroduodenal toxicity of the drug.

The aspirin targets COX-1 and 2 are associated with the upstream PI3K signaling axis, but because these enzymes are also inhibited by other NSAIDs, it is difficult to explain why the clinical effect of aspirin is specific to the drug, particularly in the context of prevention/treatment of *PIK3CA*-mutated CRC. Yet, increased generation of 15-(R)-HETE and the formation of aspirin-triggered lipoxins and resolvins distinguishes the pharmacodynamics of aspirin from that of other NSAIDs and selective COX-2 inhibitors. Moreover, as *PIK3CA* mutations, especially of the helical domain, render the enzyme independent of upstream RTK activation signals, it seems likely that the target responsible for its inhibition must be sought downstream. However, if the effect is prostaglandin-related, it is more likely connected to inhibition of cyclooxygenase expression mediated by hyperactive PI3K than to classical COX-acetylation. Nevertheless, further studies are required to decipher the importance of both COX isoforms in the context of *PIK3CA*-mutated malignancies and the effect of aspirin thereon.

**Fig. 4:**
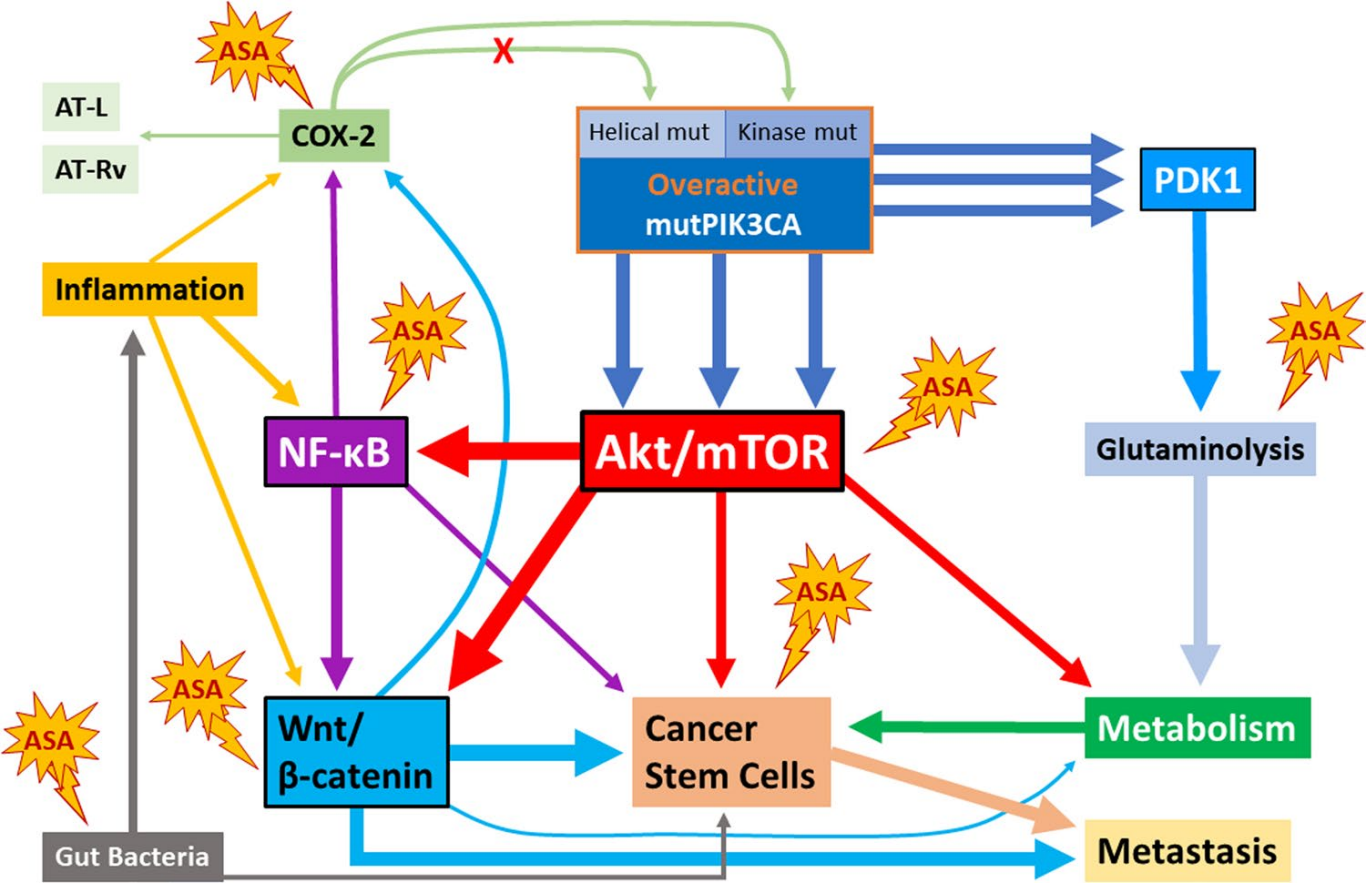
Potential key targets of aspirin in *PIK3CA*-mutated CRC. Mutations of the *PIK3CA* helical and kinase domain lead to an overactivity of PI3K (visualized by multiple arrows). Helical domain mutations decouple the enzyme from upstream signals by RTK, indicated by the red “X”, although it has to be noted that Ras-GTP activation by external signals may still be required for full PI3K activation. Kinase domain mutations are, however, independent from Ras-GTP activation. PI3K activates Akt and PDK1. mTOR is both an activator of Akt and a downstream target and, therefore, closely linked to its actions. mTOR has been identified as a direct acetylation target of aspirin (ASA), and is presumably inhibited by this. Akt is the central player connecting most pathways, however, glutaminolysis may be influenced by PDK1 Akt-independently. Aspirin has been reported to inhibit PDK1-mediated glutaminolysis by an, as yet, unresolved mechanism. Further, salicylate and aspirin have been observed to suppress NF-κB via IκB, thereby reducing chemoresistance in cancer cells. NF-κB is also involved in COX-2 expression. COX-2 has been reported to act upstream of PI3K via PGE_2_/EP4 and to modify the activity of growth factor receptors (e.g. IGFR or EGFR), inducing its own expression via a feedback loop. It is acetylated by aspirin at S516 which disables the production of PGH_2_, but enables the formation of aspirin-triggered lipoxin (AT-L) and resolvins (AT-Rv) in a transcellular fashion. In addition, salicylate may suppress the transcription of COX-2 mRNA. In addition, aspirin has been reported to inhibit Wnt/β-catenin-mediated signaling, although the exact target of aspirin in this context is unclear. Wnt/β-catenin are also substantially involved in the pathophysiology of cancer stem cells, which are highly associated with therapy-resistance and metastasis. Stemness of these cells may also be inhibited by aspirin via p300 and NANOG. NF-κB may also be involved. Gut bacteria are involved in inflammatory processes and the maintenance of intestinal homeostasis. In this context, it has been observed that aspirin can influence the growth of intestinal bacteria, possibly leading to a shift in the intestinal microbiome towards a more favorable composition. The named pathways and their potential interconnections are shown. Arrows illustrate the connections between them, arrow thickness is related to their hypothetical importance in the carcinogenesis of the PI3K pathway. The microbiome, inflammation, as well as AT-L and AT-Rv formation are depicted, despite not being directly involved in PI3K signaling. However, as they are influenced by aspirin, they might enhance the anticarcinogenic effect seen in patients with *PIK3CA*-mutated CRC

But is there only one right answer? As such, the expected multiple interactions of aspirin and its metabolite salicylate with various complex networks of signaling pathways involving PI3K imply that numerous interactions must be considered to unravel the mechanisms of aspirin-mediated inhibition of *PIK3CA*-mutated cancer. The putative major signaling pathways responsible for carcinogenesis of *PIK3CA*-mutated CRC and the interactions of aspirin with them are shown in Fig. 4. For example, inhibitory effects of aspirin on signaling networks associated with the PI3K/Akt/mTOR pathway with known oncogenic potential, such as the NF-κB, Wnt/β-catenin, and Ras/Raf/MEK/ERK pathways (the latter shown in Fig. 2) have to be mentioned. In addition, new players have entered the field, adding to the level of complexity. For instance, the influence of non-coding RNAs, metabolism, gut microbiome, and CSCs on the development of CRC, as well as the reciprocal interactions of these factors with aspirin and *PIK3CA* mutations in the context of CRC, has long been overlooked and show promising potential in providing answers and novel treatment targets. Last, *PIK3CA*-independent actions of aspirin may contribute synergistically, for instance by inhibiting inflammation. Taken together, the sensitivity of *PIK3CA*-mutated cancers to aspirin has gained much attention, especially in recent years. Nevertheless, the key mechanisms of this effect are still unknown. However, deciphering the underlying mechanisms is of great importance, in particular with regard to identifying patients who will benefit from treatment or chemoprevention with aspirin. Further interesting research on this topic can, therefore, be expected in the coming years.

## Data Availability

Data sharing is not applicable to this article because no new data were created or analyzed in this study.

## Abbreviations

4E-BP1: Eukaryotic translation initiation factor 4E-binding protein 1
5-FU: 5-Fluorouracil
ABD: Adaptor binding domain
AMPK: Adenosine monophosphate–activated protein kinase
APC: Adenomatous polyposis coli
ASA: Acetylsalicylic acid
ASCT2: Alanine, serine, cysteine transporter 2, Neutral amino acid transporter B(0) (= SLC1A5)
ATF4: Activating transcription factor 4
AT-L: Aspirin-triggered lipoxins
ATP: Adenosine triphosphate
AT-Rv: Aspirin-triggered resolvins
BAD: Bcl-2 associated death promoter
BH domain: Breakpoint-cluster region homology domain (= RhoGAP domain)
BIM: Bcl-2-like protein
circRNA: Circular RNA
COX: Cyclooxygenases
CRC: Colorectal cancer
CSC: Cancer stem cell
DVL: Dishevelled
EGF: Epidermal growth factor
EGFR: Epidermal growth factor receptor
eIF4E: Eukaryotic translation initiation factor 4E
EMT: Epithelial-mesenchymal transition
eNOS: Endothelial nitric oxide synthase (= NOS3)
ERK: Extracellular signal-regulated kinase
FOXO: Forkhead box protein O
G6PD: Glucose-6-phosphate dehydrogenase
GLUT1: Glucose transporter 1
GPT2: Glutamate pyruvate transaminase 2
GSK3: Glycogen synthase kinase 3
HETE: Hydroxyeicosatetraenoic acid
HIF-1α: Hypoxia-inducible factor 1 *α*
HK2: Hexokinase 2
I/n/cSH2: Inter/N-terminal/C-terminal Src homology 2 domain
IFN-γ: Interferon-γ
IGF: Insulin-like growth factor
IGFR: Insulin-like growth factor 1 receptor
IκB: IκB protein
IκBα: NFKB inhibitor alpha
IKK-β: IκB kinase-beta
ILβ / 6: Interleukin 1β / 6
iNOS: Inducible nitric oxide synthase (= NOS2)
ISC: Intestinal stem cell
KRAS: Kirsten rat sarcoma virus
lncRNA: Long noncoding RNA
LPS: Lipopolysaccharide
mCRC: Metastatic colorectal cancer
MACC1: Metastasis‑associated colon cancer 1
MEK: Mitogen-activated protein kinase kinase
miRNA: Micro RNA
mRNA: Messenger RNA
mTOR: Mechanistic target of rapamycin kinase
mTORC1 / 2: Mechanistic target of rapamycin complex 1 / 2
ncRNA: Noncoding RNA
NF-κB: Nuclear factor κ B
NO: Nitric oxide
NOS2: Nitric oxide synthase 2 (= iNOS)
NSAID: Non-steroidal anti-inflammatory drug
PDK1: Phosphoinositide-dependent kinase 1
PET: Positron emission tomography
PGE_2_: Prostaglandin E_2_
PGF_2__*α*_: Prostaglandin F_2__*α*_
PGH_2_: Prostaglandin H_2_
PGHS: Prostaglandin G/H synthase (= COX)
PGI_2_: Prostacyclin
PI3K: Phosphatidylinositol 3-kinase
PIP_2_: Phosphatidylinositol 4,5-bisphosphate
PIP_3_: Phosphatidylinositol 3,4,5-trisphosphate
PKB: Protein kinase B (= Akt)
PP2A: Protein phosphatase 2A
PPP: Pentose phosphate pathway
PtdIns(3,4,5)P_3_: Phosphatidylinositol 3,4,5-trisphosphate (= PIP_3_)
PtdIns(4,5)P_2_: Phosphatidylinositol 4,5-bisphosphate (= PIP_2_)
PTEN: Phosphatase and tensin homolog
PTGIS: Prostacyclin synthase
PTGS: Prostaglandin-endoperoxide synthase (= COX)
Ras: Rat sarcoma viral oncogene
RBD: Ras-binding domain
RHEB: Ras homolog enriched in brain
RhoGAP: Rho-GTPase-activating protein domain (= BH domain)
RSK2: Ribosomal S6 kinase 2
RTK: Receptor tyrosine kinase
S6K: Ribosomal protein S6 kinase
SGK1: Serum and glucocorticoid-regulated kinase 1
SH3: Src homology 3 domain
SLC1A5: Solute carrier family 1 member 5 (= ASCT2), Neutral amino acid transporter B(0)
TBXAS1: Thromboxane A synthase 1
TNFα: Tumor necrosis factor α
TCA cycle: Tricarboxylic acid cycle
TP53: Tumor protein 53
TSC1/2: Tuberous sclerosis 1/2
TxA_2_: Thromboxane A_2_
UTR: Untranslated region
VEGF-A: Vascular endothelial growth factor A
